# Maternal psychological stress during pregnancy and newborn telomere length: a systematic review and meta-analysis

**DOI:** 10.1186/s12888-023-05387-3

**Published:** 2023-12-15

**Authors:** Reza Moshfeghinia, Ali Torabi, Sara Mostafavi, Shiva Rahbar, Mohammad Sanyar Moradi, Erfan Sadeghi, Jennifer Mootz, Hossein Molavi Vardanjani

**Affiliations:** 1 Student Research Committee, Shiraz University of Medical Sciences, Shiraz, Iran; 2 MD-MPH Department, School of Medicine, Shiraz University of Medical Sciences, Shiraz, Iran; 3 Research Center for Psychiatry and Behavioral Sciences, Shiraz University of Medical Sciences, Shiraz, Iran; 4 Islamic Azad University, Shiraz Branch, Shiraz, Iran; 5 Research Consultation Center (RCC), Shiraz University of Medical Sciences, Shiraz, Iran; 6 Department of Psychiatry, Columbia University, New York, NYC USA; 7 Research Center for Traditional Medicine and History of Medicine, School of Medicine, Shiraz University of Medical Sciences, Shiraz, Iran, Shiraz, Iran

**Keywords:** Telomere shortening, Telomere, Maternal stress, Pregnancy, Newborn

## Abstract

**Introduction:**

Telomeres protect the ends of chromosomes, and shorter leukocyte telomeres are associated with major group diseases. Maternal psychological stress may be related to the shortening of telomeres in infants. This systematic review and meta-analysis set out to consolidate the varying effect sizes found in studies of maternal psychological stress and telomere length (TL) in newborns and identify moderators of the relationship between stress during pregnancy and newborn TL.

**Methods:**

Our systematic review was registered in Prospero. Six databases (PubMed, Scopus, Embase, PsycINFO, Web of Science, and CINAHL Complete) were searched for records in English from inception to February 10, 2023. Observational studies were included that measured the relationship of psychological stress of the mother during pregnancy on the TL of the newborn. The Newcastle–Ottawa quality assessment scale was used to assess the quality of the included studies. A random-effect model was selected. Statistical analysis performed by Stata software version 17.

**Results:**

Eight studies were included for qualitative and four for quantitative analysis. There was an inverse statistically significant relationship between maternal stress and newborn TL; A one score increase in maternal psychological stress resulted in a 0.04 decrease in the TL of the newborn (*B* = *-0.04, 95% CI* = *[-0.08, 0.00], p* = *0.05*). Selectivity analysis showed that the pooled effect size was sensitive to one study; After removing this study, the pooled effect size remained significant (*B* = *-0.06, 95% CI* = *[-0. 10, -0.02], p* < *0.001).*

**Conclusion:**

Physiological and environmental factors can significantly affect the TL of newborns. Our results support a significant impact of maternal psychological stress on the TL of a newborn. This association demonstrates the significance of stress in influencing the telomere length, which can be a contributing factor in the infant’s future. Therefore, recognizing this association is crucial for understanding and addressing potential health risks and necessitates the need for additional future studies to validate our findings.

**Supplementary Information:**

The online version contains supplementary material available at 10.1186/s12888-023-05387-3.

## Introduction

Telomeres are DNA–protein structures at the end of chromosomes that protect the terminal regions from degradation and intrachromosomal fusion [1]. The end replication problem results from DNA polymerase's inability to completely replicate the ends, which causes telomeres to shorten with each replication cycle in somatic cells [2]. Telomere length (TL) shortening is not only a biomarker but also appears to be a causal factor in various physical and mental illnesses and mortality [3]. Shorter TL has been linked to major group diseases such as chronic obstructive pulmonary disease (COPD) and idiopathic pulmonary fibrosis [4], infections [5], autoimmune diseases [6], some types of cancer [7], diabetes and metabolic syndrome [8], cardiovascular diseases [9] and psychological disorders [10].

It is hypothesised that TL at birth can significantly predict TL later in life [11]. Like many other situations, genetics and environmental variables affect infants’ TL; hereditability accounts for 70% of the variances in TL [12]. Environmental factors can also affect TL. Telomeres are highly vulnerable to impairment through alkylation, ultraviolet irradiation, and oxidative stress [13]. Numerous aspects of a person's lifestyle, including physical activity, smoking, and sleep, are associated with TL. One of the most critical health issues is psychological stress [14].

Stress is a process resulting in both psychological demands and biological changes that could put a burden on someone. Chronic psychological stress at high levels has been linked to a variety of diseases and unhealthy situations, including tumor growth [15], metabolic syndrome [16], cardiovascular diseases [17], and many others. Stress and illness have a complicated relationship. Individuals differ in their susceptibility to stress. A stressful incident that causes disease in one person might not in another. To manifest as a disease, events must interact with various underlying causes [18].

Stress has a known effect on the biology of telomeres. Research has demonstrated that cumulative exposure to oxidative stress speeds up telomere erosion [13]. Furthermore, an inverse correlation between TL and an inflammatory stress response has been observed [19]. Adversity-induced stress may be responsible for accelerated early-onset telomere shortening. Psychological stress is one predictor of lifelong TL [20]. The first observations showed that women with the highest levels of perceived stress have shorter telomeres than women with less stress. This shortness is equivalent to a decade of old age [21]. First-ever research by Entringer et al. suggests that maternal psychological stress during pregnancy may be linked to reduced TL in neonates at birth [22]. Maternal stress induces the release of hormones, such as cortisol and placental corticotropin-releasing hormone (CRH), as well as mediators of inflammatory and oxidative stress. These hormones and mediators enter the fetal circulation and cause changes in the placental metabolism [23]. Other research was conducted in various populations using comparatively similar methods. Some results calculated different effects, such as a study that did not repeat this inverse effect [24]. The studies' inconsistent findings led us to identify the need for a comprehensive synthesis of the literature. In this study, we focused on maternal psychological stress during pregnancy and its relation to newborn TL.

This article aims to systematically review and synthesize the evidence from the most recent literature (indexed up to February 10, 2023) and conduct a meta-analysis. To advance clinical practice and the design of subsequent investigations, we aimed to determine the impact of self-reported maternal stress on newborns’ TL. There are reviews that measure the effect of adverse childhood experiences on TL, finding a negative association between them [25]. In a systematic review and meta-analysis, childhood trauma was significantly associated with shorter TL [26]. The relationship between shorter TL and several types of stress, such as chronic social stress, post-traumatic stress disorder, and preterm infant stress have been investigated in other reviews [26–28]. To our knowledge, no published review compiles the research results in which self-reported maternal stress and newborn TL were examined.

## Methods

This systematic review and meta-analysis followed the Preferred Reporting Items for Systematic Reviews and Meta-Analyses (PRISMA) guidelines 2020 [29]. The registration number in PROSPERO is CRD4202128517.

### Search strategy

Six electric databases (PubMed, Scopus, Embase, PsycINFO, Web of Science and CINAHL Complete) were searched for records in English from inception to February 10, 2023. Searches were performed using combinations of the following keywords: “telomere” OR “telomere shortening” AND “infant” OR “newborn” OR “child” AND “mother” OR “maternal” AND “stress” OR “depress*” OR “mental”. Search didn’t Search the above words and any synonyms included in the strategy; Detailed search strategies for each database are accessible (see Supplementary Material 1). The references of included studies were also screened to identify potentially eligible articles.

### Eligibility criteria

We included observational studies that examined the effect of maternal psychological stress during pregnancy on the TL of the newborn at the time of delivery. We excluded studies for the following: (1) Studies that assessed stress before pregnancy, which could include psychological trauma from childhood or any time before pregnancy; (2) Studies that measured the TL of a newborn at a time other than at birth; (3) insufficient data to calculate the effect of maternal stress on the TL of the newborn; (4) duplicate studies or overlapping participants; (5) reviews, editorials, conference papers, case series/reports, secondary analysis or animal experiments; (6) qualitative designs.

### Study selection

Two authors (RM and AT) independently screened the titles and the abstracts of the potentially eligible studies using EndNote. Of potentially eligible studies, separate authors independently evaluated the full text. Any conflicts related to the study design or methods and the final decision to include studies among review authors were resolved in a consensus meeting. Throughout the review process, a psychiatrist (LR) provided additional support if there were questions.

### Data extraction

Two authors (RM and AT) independently extracted the information from included articles. Any disagreements were resolved by further discussion. The following general characteristics were collected: authors and year of publication, country, study type, sample size, ethnicity, male and female ratio, psychological assessment tools, source of newborn’s telomere, TL assessment tool, and main findings of the included study (Table 1).

**Table 1:** Overview of included studies

Author, year	Country	Study type	Sample size	Ethnicity	Male (%)	Psychological assessment tool	Source of infantile telomere	Telomere length assessment tool	Main results
Entringer et al., 2013 [22]	US	Cohort	27	Non-Hispanic White, African American	48	4-item pregnancy-specific stress scale	UBC	qPCR	There was a significant, linear effect of pregnancy-specific stress on newborn TL (b = -0.099, 95% CI = -0.197, -0.002, *p* = 0.04). Also, the unadjusted relationship was not significant (b = -0.062; *p* = 0.1)
Marchetto et al., 2016 [30]	US	Cohort	24	Non-Hispanic, White, African American, Hispanic,	NR	Holmes and Rahe Stress Scale	UBC	TRF	There was a significant and negative association between maternal psychological stress and TL (b = -0.005, *p* = 0.04, 95% CI = -0.009, -0.001)
Salihu et al., 2016 [23]	US	cross-sectional	71	Black, White, Hispanic	NR	Perceived Stress Scale (PSS-4)	UBC	qPCR	Mothers were divided into three groups with low (22.5%), medium (59.2%) and high (18.3%) stress levels, and higher stress duration pregnancy associated with shorter TL (*P* < 0.05)
Send et al., 2017 [31]	Germany	Cohort	319	White	48	PSS-14	UBC	qPCR	Maternal perceived stress during pregnancy was associated with TL in newborns (β = − 0.14, *p* = 0.015)
Ammala et al., 2020 [24]	Finland	Cohort	1377	White	51.7	PSS-4	UBC	qPCR	Maternal psychological stress associated with shorter TL in the newborn (b = -0.01), but there was no significant
Izano et al., 2020 [32]	US	Cohort	355	White, Hispanic, Asian, Black	NR	PSS-14	UBC	qPCR	In TMLE analyses, perceived stress was marginally associated with shorter newborn TL (b = -0.07; 95% CI = -0.15 to 0.021), but the associations were not significant after adjusting for multiple comparisons. All linear regression estimates were not statistically significant
Verner et al., 2021 [33]	Finland	Cohort	656	White	52	PSS-4	UBC	qPCR	maternal stress during pregnancy had significant and negative association with newborn TL (b = -0.079, *p* = 0.044, 95% CI = -0.155, -0.002). after excluding all women with obstetric conditions (hypertensive conditions, preeclampsia, and diabetes). This reduced the sample size from 656 to 366. maternal stress was inversely and non-significant associated with newborn TL [b = -0.049])
Enlow et al., 2021 [34]	US	Cohort	146	White, Black, Asian, Hispanic, Other	50.7	PSS-4	UBC	qPCR	Maternal stress was positively associated with telomere lenth of male newborn (b = 0.011, 95% CI = -0.018, 0.04) and inversely with female newborn (b = -0.011, 95% CI = -0.034, -0.013), and none of them were significant

*Abbreviations*: *NR* not reported, *UBC* umbilical cord blood, *qPCR* quantitative polymerase chain reaction, *PSS* Perceived Stress Scale, *TRF* Telomere Restriction Fragment

### Quality assessment

We used the Newcastle–Ottawa Quality Assessment Scale to evaluate the risk of bias in the included cohort and cross-sectional studies. Cohort studies were classified as having a low (≥ 7 stars), moderate (5–6 stars), or high risk of bias (≤ 4 stars), with an overall quality score of 9 stars and cross-sectional studies were classified as having a low (≥ 7 stars), moderate (5–6 stars), or high risk of bias (≤ 4 stars).

Two investigators (RM and AT) conducted quality assessment independently, and discrepancies were solved through discussion and consensus, including a third investigator (HM), if needed.

### Quantitative analysis

To obtain the final effect size, unstandardized beta and standard error (SE) statistics were used. For removing the effect of the mother's stress measurement tool and the newborn’s TL measurement tool, transformations were used. Also, For Enlow et al. [34] data was reported separately based on gender of infants, which we analyzed as two separate populations. A random effects model (maximum–likelihood and restricted maximum–likelihood model) was used to pool the extracted regression coefficients. Heterogeneity between the studies was evaluated by using the chi-square test and I square statistic. Publication bias was assessed by using Begg and Egger tests. A subgroup analysis was performed to estimate the pooled effect of telomere and psychological assessment tools. A sensitivity analysis was also carried out to test the robustness of the pooled effect size. All analyses were performed in Stata software (version 17, Stata Corporation, College Station, Texas, USA). *P*-values less than 0.05 were considered statistically significant.

## Results

### Selection of studies

**Fig. 1:**
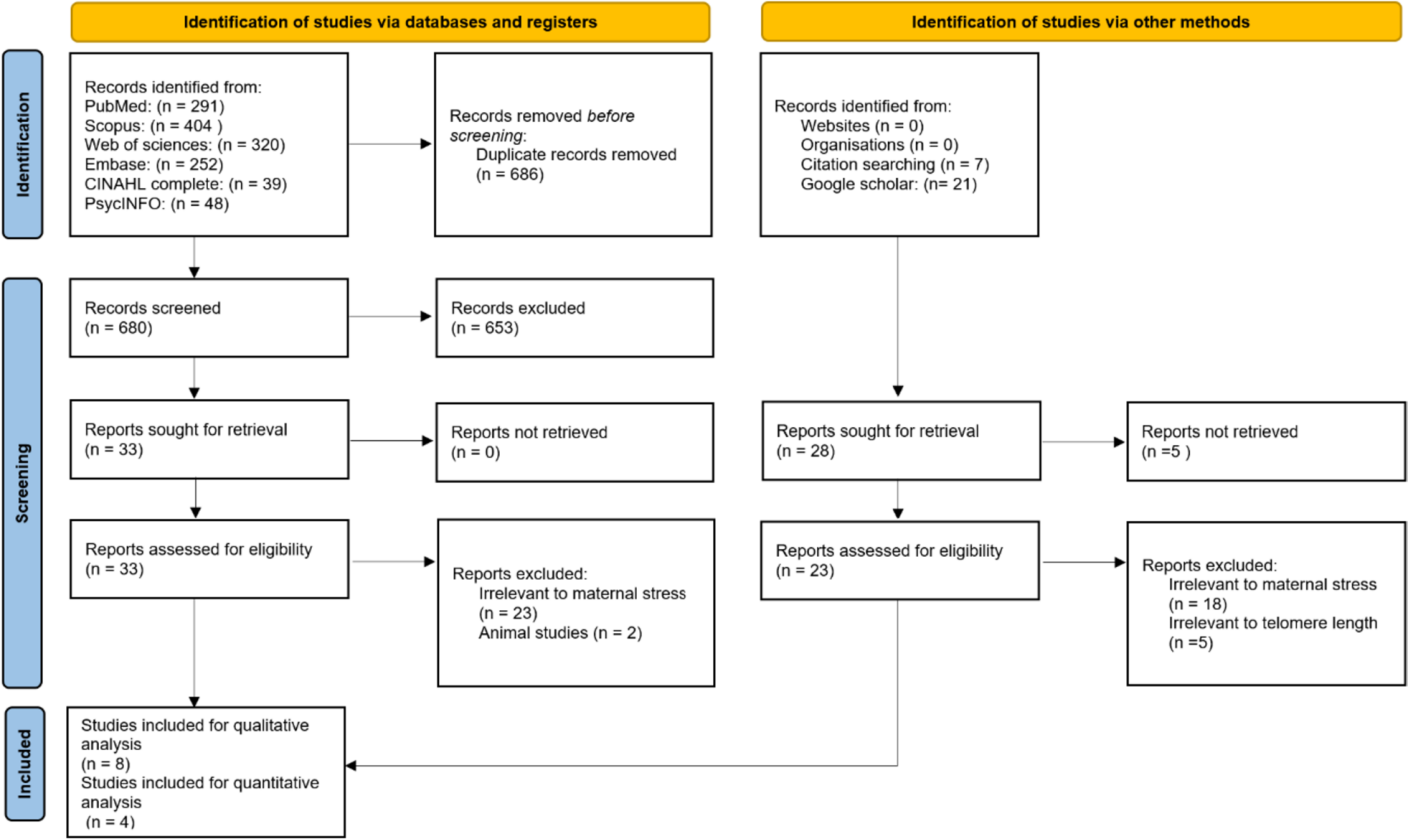
The Preferred Reporting Items for Systematic Reviews and Meta-Analysis (PRISMA) flow diagram of search results

Figure 1 depicts the PRISMA flow diagram. The search criteria initially yielded 1354 articles. We manually added five articles by reviewing reference lists of retrieved articles and seven articles by searching grey literature using Google Scholar. EndNote removed 680 duplicates, and 653 articles were excluded after screening the title and abstract. Thirty-three articles were potentially relevant to our systematic review based on the eligibility criteria. After full text evaluation, 25 articles were excluded, leaving eight remaining articles. Of those, we systematically reviewed eight studies and conducted a meta-analysis with four studies that contained the necessary quantitative data with which to run a meta-analysis.

### Study characteristics

The main features of the included studies are summarised in Table 1. The eight articles involved 2,955 participants with sample sizes varying from 24 to 1,377. All studies were performed in the last ten years. The four studies included for quantitative analysis contained 853 participants. Most studies (7 out of 8) were cohort, and only one was a cross sectional study. All studies were conducted in high-income countries. The three studies performed in Europe included White women only. However, the five other studies from the United States had samples that consisted of diverse ethnicities (White, Black, Hispanic, Asian and others).

All questionnaires used to measure mothers' stress during pregnancy were validated instruments. Etringer et al. [22] used the 4-item pregnancy-specific stress scale and Marchetto et al. used the Holmes and Rahe Stress Scale questionnaire. The remaining authors all used the Perceived Stress Scale (PSS) questionnaire. All studies used the infant’s Umbilical cord blood (UCB) cells for measurement. Two methods were used to measure neonatal TL: Telomere Restriction Fragment in one study and quantitative polymerase chain reaction (qPCR) in seven studies. Five studies reported a clear negative association between maternal psychological stress during pregnancy and newborn TL [22, 30, 33]. Two studies did not find a significant relationship [32]. In the study by Enlow et al., maternal stress was correlated with longer TL among male newborns [34].

### Risk of bias within studies

We evaluated the quality of all eight included studies according to the Newcastle–Ottawa Quality Assessment Scale and all were of good quality. They had a low risk of bias (≥ 7 stars), as shown in Table 2.

**Table 2:** Risk of bias and quality of included studies assessed by Newcastle–Ottawa Quality Assessment Scale (NOS)

The first author	Selection	Comparability	Outcome	Quality score	Risk of bias	Quality
Entringer et al., 2013 [22]	4	3	3	9	Low	Good
Marchetto et al., 2016 [30]	4	3	3	9	Low	Good
Salihu et al., 2016 [23]^a^	4	1	3	8	Low	Good
Send et al., 2017 [31]	4	3	3	9	Low	Good
Ammala et al., 2020 [24]	4	3	3	9	Low	Good
Izano et al., 2020 [32]	4	3	3	9	Low	Good
Verner et al., 2021 [33]	3	2	3	8	Low	Good
Enlow et al., 2021 [34]	4	3	3	9	Low	Good

^a^cross-sectional study

### Synthesis of results

#### Overall results

**Fig. 2:**
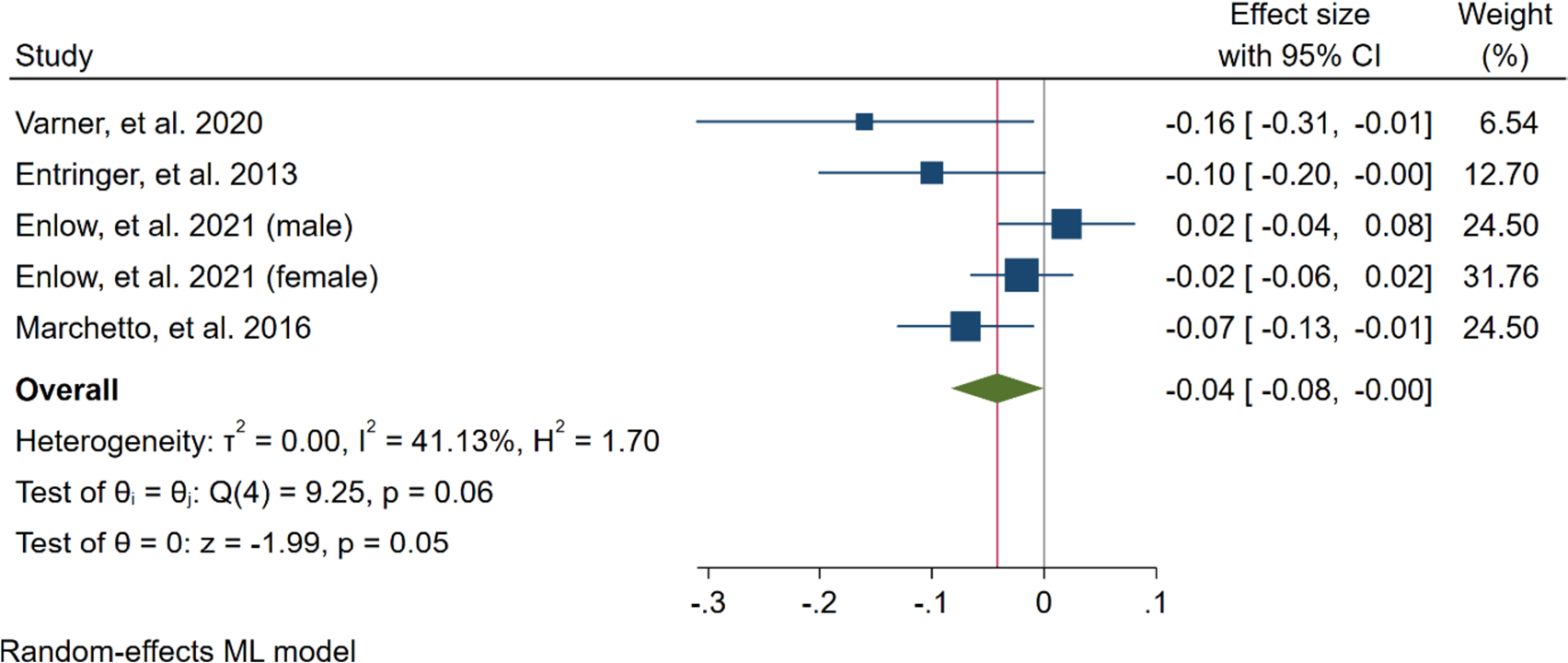
Forest plot of the overall result

**Fig. 3:**
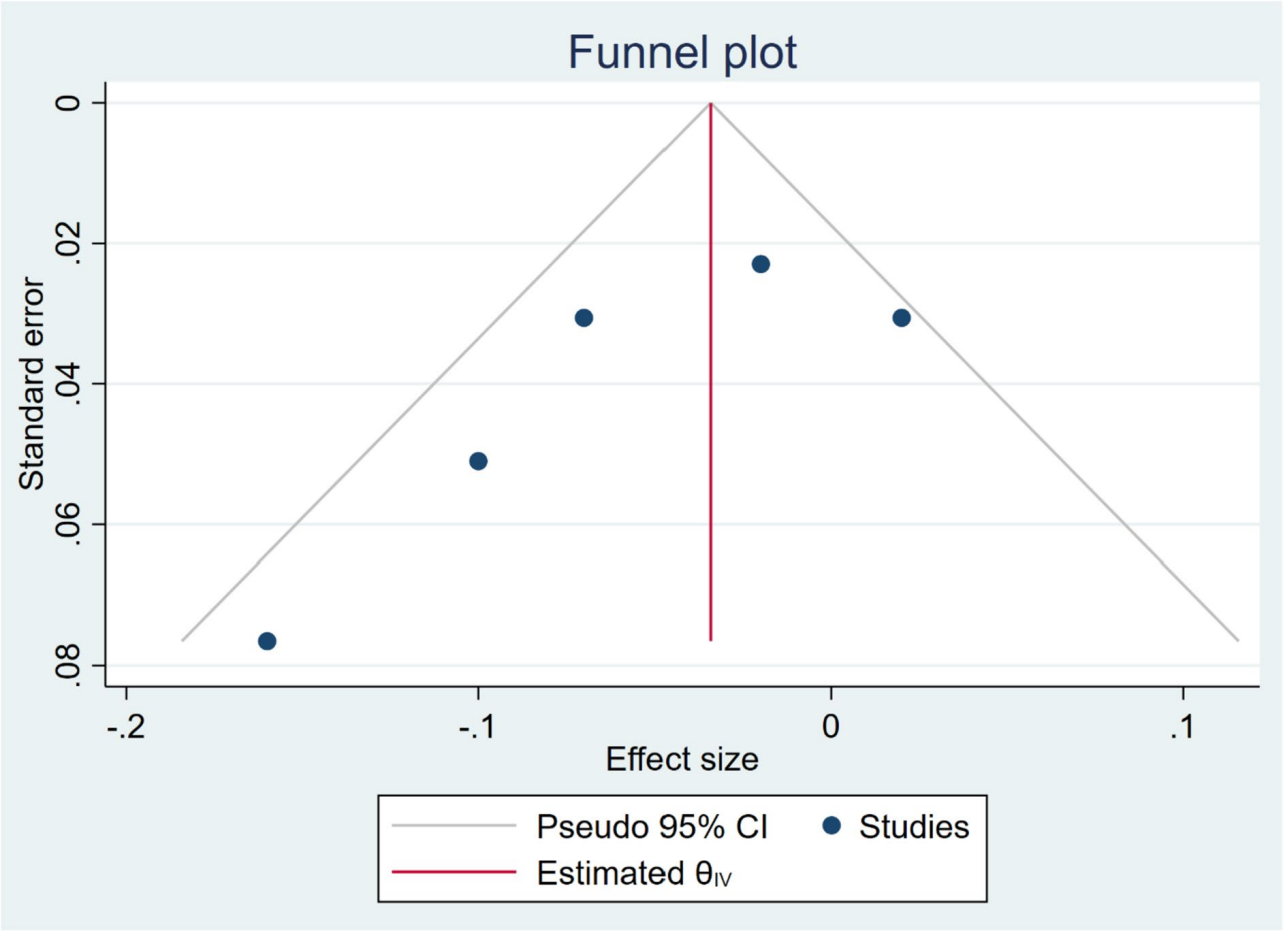
Funnel plot of the overall result

The adjusted correlation was available for four studies and a total 853 subjects. Based on the estimated pooled effect size (maximum–likelihood model), maternal-perceived stress and TL of newborn showed an inverse statistically significant relationship (B = -0.04, 95% CI = [-0.8, 0.00], *p* = 0.05). That is, a one score increase in the maternal psychological stress would result in about a 0.04 decrease in the TL of newborn. Moderate heterogeneity was found across the studies (I^2^ = 41.13%) (Fig. 2). A Funnel Plot (Fig. 3), along with Begg’s (*P* = *0.130*) and Egger’s (*P* = *0.055*) tests revealed no significant publication bias. Also, restricted maximum–likelihood model showed iverse but borderline effect (B = -0.05, 95% CI = [-0. 10, 0.00], *p* = 0.07) Supplementary Material 2, Figure S1.

#### Subgroup analysis

**Fig. 4:**
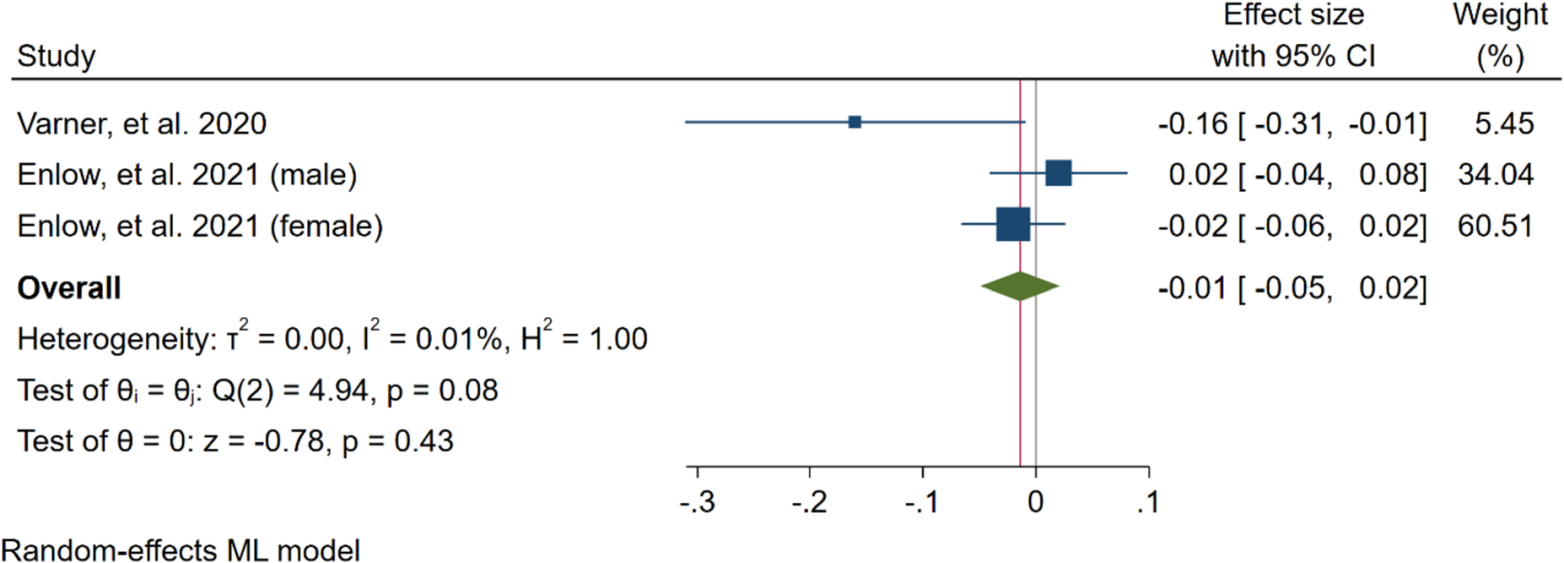
Meta-analysis of the effect of maternal psychological stress during pregnancy on newborn TL for studies used Perceived Stress Scale 4 (PSS-4) as tools for assessing psychological stress of mother

**Fig. 5:**
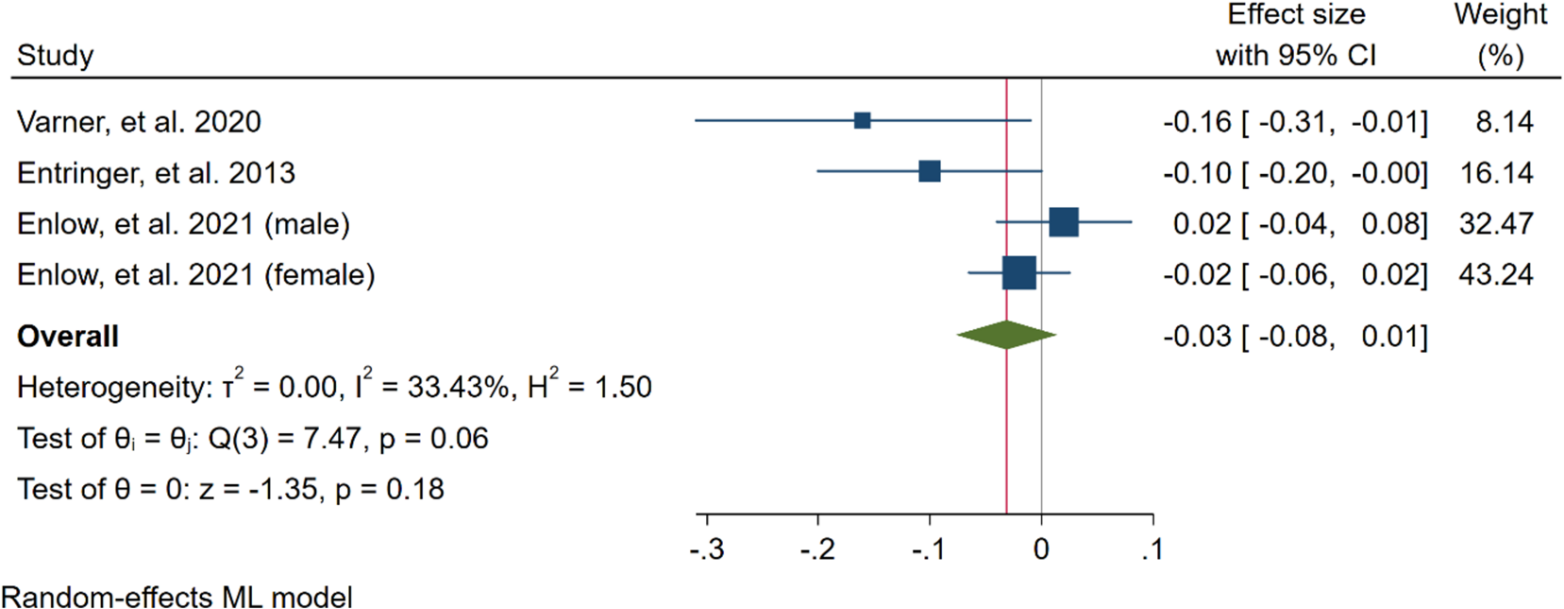
Meta-analysis of the effect of maternal psychological stress during pregnancy on newborn TL for studies used quantitative polymerase chain reaction (qPCR) for measuring TL of cord blood cells of the newborn at delivery time

Subgroup analyses were mainly processed based on the psychological assessment tool of the mother and the TL measurement tool. Analysis showed high heterogeneity and an inverse but not significant correlation between studies that used the PSS-4 (unstandardised beta = -0.01, 95% CI = -0.05 to 0.02, *p* = 0.43, I^2^ = 0.01%) and the qPCR (unstandardised beta = -0.03, 95% CI = -0.08 to 0.01, *p* = 0.18, I^2^ = 33.43%) for assessing psychological stress of mothers (Figs. 4 and 5).

#### Sensitivity analysis

**Fig. 6:**
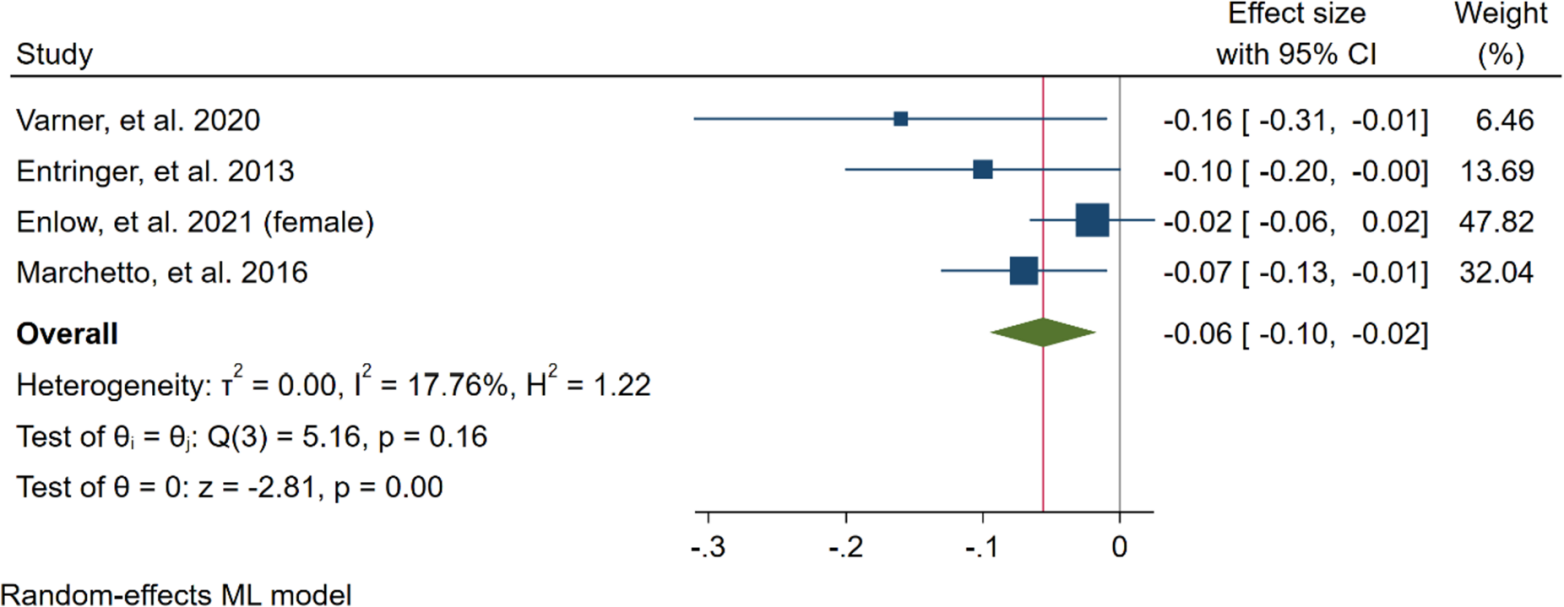
Meta-analysis of all included studies after removing one study (Sensitivity analysis)

We performed a sensitivity analysis to measure the individual impact of each study on the effect size as the main result of our mathematical model by removing one study at a time. According to our results, the study by Enlow et al. (male newborns) (2016) (Supplementary Material 2, Figure S2) deviated from the data of the other studies. After removing the male newborn data, the relation was significant (unstandardised beta = -0.06, 95% CI = -0.10 to -0.02, *p* < 0.001). Also, heterogeneity changed to low (I^2^ = 17.76%) (Fig. 6). Also, for restricted maximum–likelihood model the same study removed and the effect change to significant (unstandardised beta = -0.06, 95% CI = -0.11 to -0.02, *p* = 0.01, and, heterogeneity changed to low (I2 = 38.85%) (Supplementary Material 2, Figure S3).

## Discussion

We reviewed the relationship between maternal-perceived stress during pregnancy and the TL of newborns and identified eight studies that fulfilled the inclusion criteria.

The results of four included studies in the meta-analysis revealed that while newborn TL would reduce by around 0.04 for every point of increase of maternally perceived stress, this relation was statistically significant. Once the male newborn data was removed, the level of heterogeneity decreased from 41.13% to 17.76%, while the association remained statistically significant (unstandardised beta = -0.06, *p* < 0.001). Therefore, investigating how gender affects this relationship is necessary. For this reason, we evaluated data from Enlow er al. based on gender of infants, which we analyzed as two separate populations. 

We conducted a thorough review of eight included studies. It should be mentioned that there were some discrepancies in the findings of the included studies regarding this potential association. In five studies an inverse association between maternal-perceived stress and newborn TLwas reported [22, 23, 30, 31, 33]. Entringer et al. published the first preliminary data in humans demonstrating a link between prenatal stress exposure and shorter TL in 27 young adults. An inverse significantrelationship was reported (*β* = *-0.099; P* = *0.04*) [35]. In a cross-sectional study, Salihu et al. examined 71 women of low-socioeconomic status, indicating that this population has a higher risk of stress. They discovered an inverse significantrelationship (*P* < *0.05* in 5000 of the 5000 repetitions using repeated ANOVA) [36]. The design of the study prohibited claims about causality, so more longitudinal studies are needed. Verner’s comparably large cohort with 656 participants replicate previous reports linking maternal stress during pregnancy with offspring TL with a borderline significant relationship (*β* = *-0.079, p* = *0.044*). In Verner’s study, women had at least one risk factor for preeclampsia [31]. Early hypoperfusion or placental ischemia–reperfusion injury in preeclampsia leads to increased oxidative stress. According to Send et al., maternal perceived stress during pregnancy was significantly associated with shorter telomeres in newborns (*β* = *-0.14; P* = *0.015*) [13]. Marchetto et al. conducted a prospective cohort study of 24 mothers and found a significant negative association between maternal stress and newborn TL (*β* = *-0.463, P* = *0.04*) [13].

In the study conducted by Izano et al., maternal-perceived stress had a marginal association with shorter newborn TL; however, after adjusting for multiple comparisons, the relationship remained insignificant (*β* = *-0.07; P* = *0.29).* In addition to using a linear regression model, this study applied Targeted Minimum Loss-Based Estimation (TMLE) to evaluate the relationship between TL and maternal exposure to stress [32]. Ämmälä et al., with a sample of 1405 infants, could not confirm a correlation between maternal stress and leukocyte TL in newborns. Some possible causes were differences in the TL analysis methods between studies, variations in the statistical models, different cell populations in umbilical blood samples, and differences in measures of maternal well-being and the timing of measurements [24]. On the other hand, Enlow et al. reported an association in the opposite direction. They showed maternal stress correlated with longer TL among male newborns (*β* = *0.011*) [34]. It was the only study exploring potential sex effects on these associations.

In six studies, the PSS-4 was used to assess stress levels. PSS-4 is one of the most common tools for evaluating psychological stress. It is a valuable tool for assessing stress during pregnancy and can help clinicians obtain an accurate evaluation of the degree of maternal stress for optimising health care among pregnant women [37]. Seven studies used qPCR to determine TL. Only one study used the Telomere Restriction Fragment tool. All these measures are validated in measuring self-reported maternal stress during pregnancy. Some studies investigated the effects of gender on TL, and some further evaluated the role of gender in the association between maternal stress and TL. Izano et al. observed that a high level of maternal perceived stress was correlated with shorter TL among girls but longer among boys [32]. They stated that the difference in the association between maternal stress and TL among males and females might suggest the different influences of financial strain, unplanned pregnancy, and perceived stress; high levels of perceived stress and unplanned pregnancy were associated with shorter TL among females but longer among males. Food insecurity was strongly associated with male newborns and not female ones [32]. Verner et al. reported that the female sex was a significant predictor of TL at birth, as TL was shown to be longer in girls [33]. Ämmälä et al. found no significant difference in the association between maternal stress and TL between the sexes [24]. Entringer et al. observed no significant difference between males and females regarding TL [22]. Enlow et al. found no sex differences in TL; however, they reported that increased maternal stress predicted longer newborn TL among male newborns [34]. Sex differences in TL are controversial, but it is widely accepted that women have longer telomeres than men [38]. However, as the difference is barely seen in newborns, the effects of sex may be primarily related to different rates of telomere attrition after birth which mainly depends on estrogen. Estrogen can increase telomerase activity and, on the other hand, decrease oxidative stress [39]. Collectively, the present studies provide evidence that this relationship should be investigated.

The effect of preeclampsia on newborn TL was investigated in some included studies. Verner et al. reported no significant effect [33], while Enlow et al. reported that preeclampsia in a prior pregnancy was negatively associated with newborn telomere [34]. It has been proposed that in pregnancies complicated with preeclampsia, the length of the telomeres is significantly shorter [40]. A previous study showed shorter telomeres in trophoblast in preeclampsia with decreased expression of the human telomerase reverse transcriptase (hTERT) component of the human telomerase enzyme [41]. Another study reported a significant decrease in levels of protective proteins such as Sirtunin1 (SIRT1) in placentas from preeclampsia resulting in shorter telomeres [42].

Participants in three studies were completely white, making them non-representative samples.. In the US studies, however, samples consisted of diverse races/ethnicities. Enlow et al. and Izano et al. showed that newborns' race/ethnicity was not associated with TL [34]. However, the association between maternal stressors and TL in newborns varies by race/ethnicity; perceived stress is accompanied by shorter newborn TL among White women [32]. The impact of the maternal psychosocial condition on newborn TL is associated with sociodemographic characteristics. Different mechanisms have been suggested for this variety. Pregnant women of a racial minority are at higher risk for nutritional deficiencies, which may be associated with reduced newborn TL [34]. Race differences in newborn TL may also vary by receipt of public assistance. Infants born to black mothers have longer telomeres than those born to white mothers.This is contrary to the assumption that a mother’s social disadvantage is associated with shorter TL in offspring [43].

While the current meta-analysis is a first, the findings should be considered along with its limitations. We included only four studies in our meta-analysis; the number of studies conducted on this topic is limited. Given the limitations we had such as restricted number of studies and the presence of moderate heterogeneity, we determined that the maximum likelihood model was the most suitable one to utilize. Differences in LTL analysis methods, stress measurement tools, statistical models, and study populations across studies posed challenges in combining data and led to some limitations in the generalizability of the findings.As these kinds of research require advanced tools and precise follow-up, the existing studies have been performed in a few high-income countries. In all included studies, the measure for stress was a self-reported questionnaire. No biological marker, such as maternal or cord blood oxidative stress marker, was examined. Furthermore, there was a wide range in the number of subjects included in the studies. We could not access the full text of one study despite contacting the corresponding author three times and other authors one time. Our study exclusively focused on English-written articles. Consequently, we recommend that future studies consider incorporating articles published in other languages to enhance the representation of research within the field.

In summary, we identified and systematically reviewed 8 studies that investigated the relationship between maternal-perceived stress and newborn TL; 4 studies were included in the meta-analysis. The findings of the studies overally point to aninverse statistically significant relationship among them, which was supported by our meta-analysis.. Individuals with shortened telomeres are more susceptible to developing age-related diseases, and this association between TL and chronic diseases is mainly determined in the first decades of life. This raises concern as maternal-perceived stress during pregnancy can increase the risk for health problems in their offspring from an early age. We recommend further studies to investigate this correlation in large sample sizes and determine the exact mechanisms and risk factors for shortened telomeres in newborns of mothers who experienced high stress levels during pregnancy. Subsequent studies may measure stress using biological markers. Further research is needed to understand the complex relationship between maternal psychological stress during pregnancy and TL, which may yield deeper insights and allow us to identify and protect pregnant women from stressful situations.

## Conclusion

Telomere length varies significantly among newborns, which indicates the role of physiological and environmental factors during the fetal period. In this study, the analysis of the existing studies revealed an inverse statistically significant relationship between maternal-perceived stress and newborn TL. This research provides a better understanding of the underlying mechanisms and pathways involved. We suggest that early identification and assessment of pregnant women experiencing high levels of stress, along with health follow-ups of infants whose mothers experienced psychological stress during pregnancy, could be beneficial to support the well-being of both mothers and their infants. This systematic review and meta-analysis is a step towards implementing more studies considering some important limitations that were mentioned.

### Supplementary Information

**Additional file 1. **PRISMA checklist.

**Additional file 2: Figure S1.** Forest plot of the overall result. **Figure S2.** Sensitivity analysis of included studies. **Figure S3.** Meta-analysis of all included studies after removing one study (Sensitivity analysis).

## Data Availability

All information required is given in the text and supplementary materials, other supplementary information can be obtained upon email from the corresponding author.
