# Supplementary material for: Maternal psychological stress during pregnancy and newborn telomere length: a systematic review and meta-analysis
Source: BMC Psychiatry. 2023 Dec 15;23:947. doi: 10.1186/s12888-023-05387-3 (PMC10724935; doi:10.1186/s12888-023-05387-3)
Supplement: Supplementary file 1 — Additional file 1. PRISMA checklist. [file 12888_2023_5387_MOESM1_ESM.pdf]

Data bases

1. **PubMed**: 291 results, no similar review and systematic review

((((((((("Telomere Shortening"[Mesh]) OR "Telomere"[Mesh])) OR (telomer*[Title/Abstract])) OR (telomere[Title/Abstract])) OR (telomere shortening[Title/Abstract])) AND ((((((((((((("Infant, Newborn"[Mesh]) OR "Child, Preschool"[Mesh]) OR "Child"[Mesh]) OR "Infant"[Mesh]) OR "Fetus"[Mesh]) OR (newborn[Title/Abstract])) OR (child*[Title/Abstract])) OR (preschool child[Title/Abstract])) OR (infan*[Title/Abstract])) OR (neonate[Title/Abstract])) OR (fetus[Title/Abstract])) OR (baby[Title/Abstract])) OR (fetal[Title/Abstract]))) AND (((((((((((((((((((((stress*[Title/Abstract]) OR (mental[Title/Abstract])) OR (psycho*[Title/Abstract])) OR (psychosocial[Title/Abstract])) OR (Resilience[Title/Abstract])) OR (violence[Title/Abstract])) OR (tension[Title/Abstract])) OR (emotion*[Title/Abstract])) OR (pressure[Title/Abstract])) OR (anxiety[Title/Abstract])) OR (Nervousness[Title/Abstract])) OR (Anxiousness[Title/Abstract])) OR (Hypervigilance[Title/Abstract])) OR (Angst[Title/Abstract])) OR (trauma[Title/Abstract])) OR (distress[Title/Abstract])) OR (sadness[Title/Abstract])) OR (Unhappiness[Title/Abstract])) OR (tragic[Title/Abstract])) OR (behavior[Title/Abstract])) OR (((((((((((((((((((("Occupational Stress"[Mesh]) OR "Stress, Psychological"[Mesh]) OR "Stress, Physiological"[Mesh]) OR "Stress Disorders, Traumatic, Acute"[Mesh]) OR "Stress Disorders, Traumatic"[Mesh]) OR "Mental Health"[Mesh]) OR "Mental Disorders"[Mesh]) OR "Psychotic Disorders"[Mesh]) OR "Psychophysiologic Disorders"[Mesh]) OR "Psychology, Adolescent"[Mesh]) OR "Resilience, Psychological"[Mesh]) OR "Psychomotor Disorders"[Mesh]) OR "Psychological Distress"[Mesh]) OR "Psychological Trauma"[Mesh]) OR "Psychosocial Deprivation"[Mesh]) OR "Violence"[Mesh]) OR "Emotional Abuse"[Mesh]) OR "Anxiety"[Mesh]) OR "Anxiety Disorders"[Mesh]) OR "Sadness"[Mesh]))) AND (((((((((maternal[Title/Abstract]) OR (mother*[Title/Abstract])) OR (prenatal[Title/Abstract])) OR (motherly[Title/Abstract])) OR (antenatal[Title/Abstract])) OR (pregnancy[Title/Abstract])) OR (Gestation*[Title/Abstract])) OR (motherhood[Title/Abstract])) OR (pregnant[Title/Abstract])) OR (((("Mothers"[Mesh]) OR "Mother-Child Relations"[Mesh]) OR "Pregnancy"[Mesh]) OR "Pregnant Women"[Mesh]))

2. **Scopus**: 404 (8 sec) results, no similar review and systematic review

(TITLE-ABS-KEY(telomere) OR TITLE-ABS-KEY(telomere shortening) OR TITLE-ABS-KEY(telomere*)) AND (TITLE-ABS-KEY(neonate) OR TITLE-ABS-KEY(child*) OR TITLE-ABS-KEY(newborn) OR TITLE-ABS-KEY(infan*) OR TITLE-ABS-KEY(fetal) OR TITLE-ABS-KEY(preschool child) OR TITLE-ABS-KEY(baby) OR TITLE-ABS-KEY(fetus)) AND (TITLE-ABS-KEY(maternal) OR TITLE-ABS-KEY(mother*) OR TITLE-ABS-KEY(motherhood) OR TITLE-ABS-KEY(motherly) OR TITLE-ABS-KEY(prenatal) OR TITLE-ABS-KEY(antenatal) OR TITLE-ABS-KEY(pregnancy) OR TITLE-ABS-KEY(gestation*) OR TITLE-ABS-KEY(pregnant)) AND (TITLE-ABS-KEY(stress*) OR TITLE-ABS-KEY(psychosocial) OR TITLE-ABS-KEY(mental) OR TITLE-ABS-KEY(psycho*) OR TITLE-ABS-KEY(Resilience) OR TITLE-ABS-KEY(violence) OR TITLE-ABS-KEY(tension) OR TITLE-ABS-KEY(emotion*) OR TITLE-ABS-KEY(pressure) OR TITLE-ABS-KEY(anxiety) OR TITLE-ABS-KEY(Nervousness) OR TITLE-ABS-KEY(Anxiousness) OR TITLE-ABS-KEY(Hypervigilance) OR TITLE-ABS-KEY(Angst) OR TITLE-ABS-KEY(trauma) OR TITLE-ABS-KEY(distress) OR TITLE-ABS-KEY(sadness) OR TITLE-ABS-KEY(Unhappiness) OR TITLE-ABS-KEY(tragic) OR TITLE-ABS-KEY(behavior))

3. **PsycINFO**: 48 results, no similar review and systematic review

#1: TI telomere OR TI telomere* OR TI telomere shortening OR AB telomere OR AB telomere* OR AB telomere shortening

#2: TI newborn OR TI child* OR TI preschool child OR TI infan* OR TI neonate OR TI fetus OR TI baby OR TI fetal OR AB newborn OR AB child* OR AB preschool child OR AB infan* OR AB neonate OR AB fetus OR AB baby OR AB fetal

3#: TI maternal OR TI mother* OR TI prenatal OR TI motherly OR TI antenatal OR TI pregnancy OR TI Gestation* OR TI motherhood OR TI pregnant OR AB maternal OR AB mother* OR AB prenatal OR AB motherly OR AB antenatal OR AB pregnancy OR AB Gestation* OR AB motherhood OR AB pregnant

#4: TI stress* OR TI mental OR TI psycho* OR TI psychosocial OR TI Resilience OR TI violence OR TI tension OR TI emotion* OR TI pressure OR TI anxiety OR TI Nervousness OR TI Anxiousness OR TI Hypervigilance OR TI Angst OR TI trauma OR TI distress OR TI sadness OR TI Unhappiness OR TI tragic OR TI behavior OR AB stress* OR AB mental OR AB psycho* OR AB psychosocial OR AB Resilience OR AB violence OR AB tension OR AB emotion* OR AB pressure OR AB anxiety OR AB Nervousness OR AB Anxiousness OR AB Hypervigilance OR AB Angst OR AB trauma OR AB distress OR AB sadness OR AB Unhappiness OR AB tragic OR AB behavior

Search strategy: #4 AND #3 AND #2 AND #1

4. **Web of science**: 320 results, no similar review and systematic review

#1: TS=(telomere OR telomere shortening OR telomere*)

#2: TS=(newborn OR fetal OR child* OR infan* OR neonate OR baby OR preschool child OR fetus)

#3: TS=(maternal OR mother* OR prenatal OR motherly OR antenatal OR pregnancy OR Gestation* OR motherhood OR pregnant)

#4: TS=(stress* OR mental OR psychosocial OR psycho* OR Resilience OR violence OR tension OR emotion* OR pressure OR anxiety OR Nervousness OR Anxiousness OR Hypervigilance OR Angst OR trauma OR distress OR sadness OR Unhappiness OR tragic OR behavior)

Search strategy: #4 AND #3 AND #2 AND #1

5. **CINAHL**: 39 results, no similar review and systematic review

#1: TI telomere OR TI telomere* OR TI telomere shortening OR AB telomere OR AB telomere* OR AB telomere shortening

#2: TI newborn OR TI child* OR TI preschool child OR TI infan* OR TI neonate OR TI fetus OR TI baby OR TI fetal OR AB newborn OR AB child* OR AB preschool child OR AB infan* OR AB neonate OR AB fetus OR AB baby OR AB fetal

3#: TI maternal OR TI mother* OR TI prenatal OR TI motherly OR TI antenatal OR TI pregnancy OR TI Gestation* OR TI motherhood OR TI pregnant OR AB maternal OR AB mother* OR AB prenatal OR AB motherly OR AB antenatal OR AB pregnancy OR AB Gestation* OR AB motherhood OR AB pregnant

#4: TI stress* OR TI mental OR TI psycho* OR TI psychosocial OR TI Resilience OR TI violence OR TI tension OR TI emotion* OR TI pressure OR TI anxiety OR TI Nervousness OR TI Anxiousness OR TI Hypervigilance OR TI Angst OR TI trauma OR TI distress OR TI sadness OR TI Unhappiness OR TI tragic OR TI behavior OR AB stress* OR AB mental OR AB psycho* OR AB psychosocial OR AB Resilience OR AB violence OR AB tension OR AB emotion* OR AB pressure OR AB anxiety OR AB Nervousness OR AB Anxiousness OR AB Hypervigilance OR AB Angst OR AB trauma OR AB distress OR AB sadness OR AB Unhappiness OR AB tragic OR AB behavior

Search strategy: #4 AND #3 AND #2 AND #1

6. **Embase:** 252 results, no similar review and systematic review

(telomere:ab,ti OR 'telomere shortening':ab,ti OR telomere*:ab,ti) AND (infan*:ab,ti OR fetal:ab,ti OR child*:ab,ti OR fetus:ab,ti OR newborn:ab,ti OR neonate:ab,ti OR baby:ab,ti OR 'preschool child':ab,ti) AND (maternal:ab,ti OR mother*:ab,ti OR prenatal:ab,ti OR motherly:ab,ti OR antenatal:ab,ti OR pregnancy:ab,ti OR Gestation*:ab,ti OR motherhood:ab,ti OR pregnant:ab,ti) AND (stress*:ab,ti OR psychosocial:ab,ti OR mental:ab,ti OR psycho*:ab,ti OR Resilience:ab,ti OR violence:ab,ti OR tension:ab,ti OR emotional:ab,ti OR pressure:ab,ti OR anxiety:ab,ti OR Nervousness:ab,ti OR Anxiousness:ab,ti OR Hypervigilance:ab,ti OR Angst:ab,ti OR trauma:ab,ti OR distress:ab,ti OR sadness:ab,ti OR Unhappiness:ab,ti OR tragic:ab,ti OR behavior:ab,ti)
