# Supplementary material for: Maternal psychological stress during pregnancy and newborn telomere length: a systematic review and meta-analysis
Source: BMC Psychiatry. 2023 Dec 15;23:947. doi: 10.1186/s12888-023-05387-3 (PMC10724935; doi:10.1186/s12888-023-05387-3)
Supplement: Supplementary file 2 — Additional file 2: Figure S1. Forest plot of the overall result. Figure S2. Sensitivity analysis of included studies. Figure S3. Meta-analysis of all included studies after removing one study (Sensitivity analysis). [file 12888_2023_5387_MOESM2_ESM.pdf]

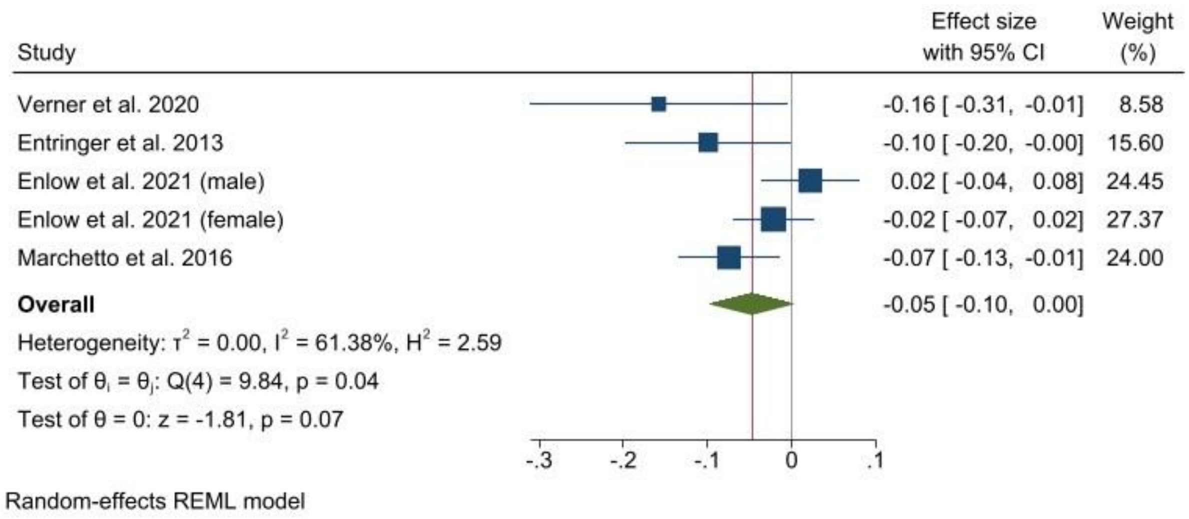


Figure S1. Forest plot of the overall result

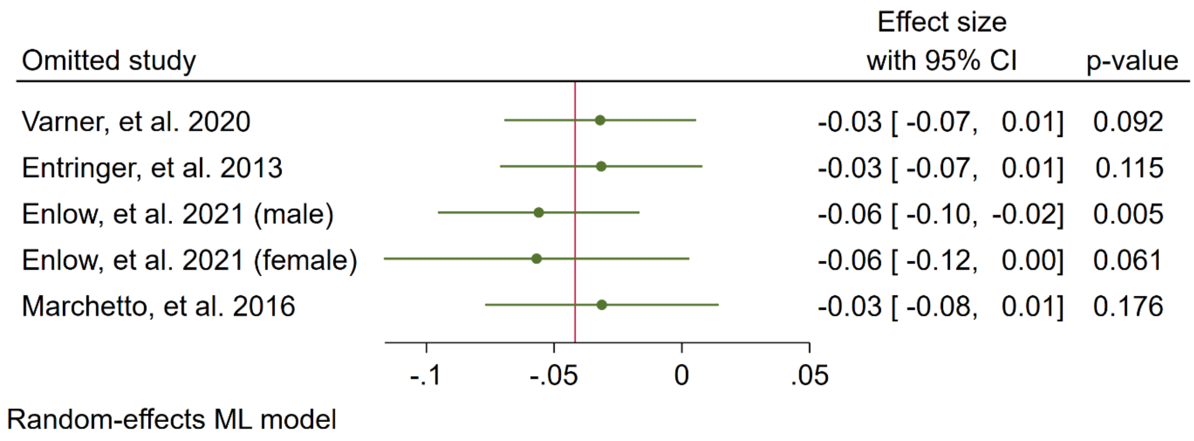


Figure S2. Sensitivity analysis of included studies

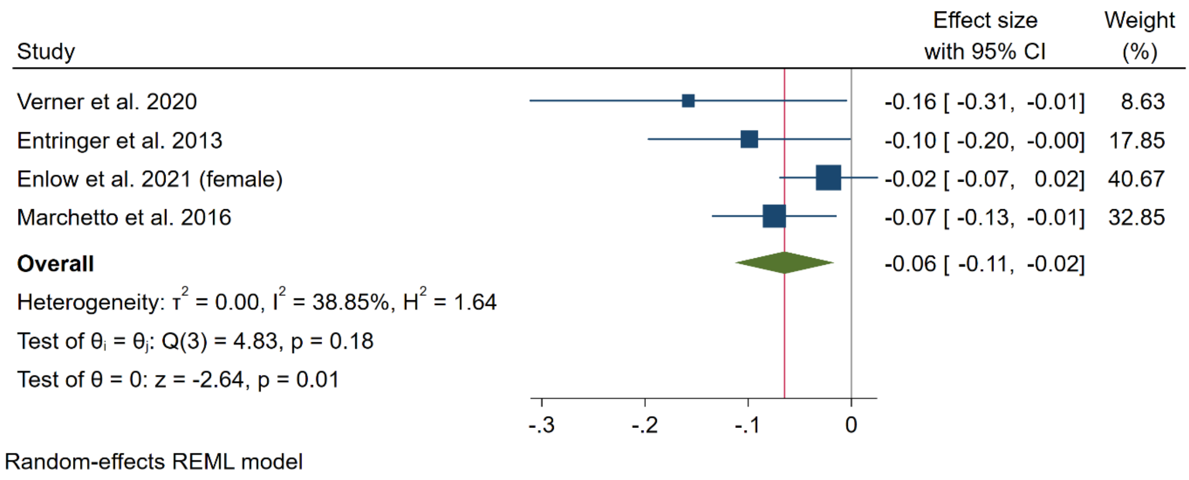


Figure S3. Meta-analysis of all included studies after removing one study (Sensitivity analysis).
